# Extracellular matrix induced by steroids and aging through a G-protein-coupled receptor in a *Drosophila* model of renal fibrosis

**DOI:** 10.1242/dmm.041301

**Published:** 2020-06-24

**Authors:** Wenjing Zheng, Karen Ocorr, Marc Tatar

**Affiliations:** 1 Department of Ecology and Evolutionary Biology, Division of Biology and Medicine, Brown University, Providence RI 02912, USA; 2 Development, Aging and Regeneration Program, SBP Medical Discovery Institute, La Jolla, CA 92037, USA

**Keywords:** Fibrosis, Aldosterone, G-protein-coupled receptor, DopEcR, Aging, Ecdysone

## Abstract

Aldosterone is produced by the mammalian adrenal cortex to modulate blood pressure and fluid balance; however, excessive, prolonged aldosterone promotes fibrosis and kidney failure. How aldosterone triggers disease may involve actions independent of its canonical mineralocorticoid receptor. Here, we present a *Drosophila* model of renal pathology caused by excess extracellular matrix formation, stimulated by exogenous aldosterone and by insect ecdysone. Chronic administration of aldosterone or ecdysone induces expression and accumulation of collagen-like Pericardin in adult nephrocytes – podocyte-like cells that filter circulating hemolymph. Excess Pericardin deposition disrupts nephrocyte (glomerular) filtration and causes proteinuria in *Drosophila*, hallmarks of mammalian kidney failure. Steroid-induced Pericardin production arises from cardiomyocytes associated with nephrocytes, potentially reflecting an analogous role of mammalian myofibroblasts in fibrotic disease. Remarkably, the canonical ecdysteroid nuclear hormone receptor, Ecdysone receptor (EcR), is not required for aldosterone or ecdysone to stimulate Pericardin production or associated renal pathology. Instead, these hormones require a cardiomyocyte-associated G-protein-coupled receptor, Dopamine-EcR (DopEcR), a membrane-associated receptor previously characterized in the fly brain to affect behavior. DopEcR in the brain is known to affect behavior through interactions with the *Drosophila* Epidermal growth factor receptor (Egfr), referred to as dEGFR. Here, we find that the steroids ecdysone and aldosterone require dEGFR in cardiomyocytes to induce fibrosis of the cardiac-renal system. In addition, endogenous ecdysone that becomes elevated with age is found to foster age-associated fibrosis, and to require both cardiomyocyte DopEcR and dEGFR. This *Drosophila* renal disease model reveals a novel signaling pathway through which steroids may modulate mammalian fibrosis through potential orthologs of DopEcR.

## Abstract (precis)

**Summary:** *Drosophila* kidney function in impaired by the steroid hormone ecdysone and by human aldosterone, and when ecdysone increases with age. Steroids induce fibrosis in the fly kidney by signaling through the Dopamine-Ecdysone receptor.

## INTRODUCTION

Aldosterone is a primary renal regulator of Na^+^ and K^+^ homeostasis, but when chronically elevated as in diabetes and primary aldosteronism (Nagase and Fujita, 2011), aldosterone promotes kidney interstitial fibrosis and glomerulosclerosis (Azibani et al., 2013; Ibrahim and Hostetter, 2003; Brown, 2013). These events are preceded by elevated inflammation through monocytes and macrophage infiltration followed by proliferation of myofibroblasts that secrete fibrinogen, collagens and elastins. Aldosterone increases reactive oxygen species to induce profibrotic factors such as transforming growth factor-β1 (TGF-β1), plasminogen activator inhibitor-1 and endothelin-1 (Brown, 2013). TGF-β1 contributes to fibrosis by activating myofibroblasts (Barnes and Gorin, 2011), as well as through suppressing matrix metalloproteinases, which can further promote excess extracellular matrix (ECM) (Zhao et al., 2013). Aldosterone affects these processes through its interaction with the mineralocorticoid nuclear hormone receptor (MR), as inferred from studies in which blockade of MR activity prevents aldosterone-associated inflammatory and fibrotic outcomes (Tesch and Young, 2017; Ibarrola et al., 2018; Montes-Cobos et al., 2015).

Many data also suggest that aldosterone contributes to fibrosis through rapid signaling independent of MR (Brown, 2013). Aldosterone enhances TGF-β1 expression and fibrosis in part through stimulation of extracellular signal-regulated kinase 1/2 (ERK1/2; also known as MAPK3/1) (Han et al., 2009; Fu et al., 2012; Min et al., 2005), while aldosterone fosters hypertrophy in cardiomyocytes through action on ERK5 (also known as MAPK7) and PKC (Araujo et al., 2016). In addition, aldosterone effectively induces calcium influx in fibroblasts derived from MR-deficient mice (Haseroth et al., 1999). Angiotensin receptors crosstalk with MR to modulate NF-κB in vascular smooth muscle cells (VSMCs) stimulated with aldosterone (Lemarie et al., 2009), suggesting that aldosterone can in part act through G-protein-coupled receptors (GPCRs). With considerable debate, GPER1 has been proposed as an alternative GPCR for aldosterone (Wendler et al., 2012; Feldman et al., 2016; Ren et al., 2014; Brem et al., 2011). In VSMCs, aldosterone was seen to activate PI3 kinase and ERKs through both GPER1 and MR (Gros et al., 2011). Emerging evidence, however, shows that 17β-estradiol is the steroid agonist of GPER1 (Evans et al., 2016; Barton and Meyer, 2015; Cheng et al., 2014), and no pharmacological evidence demonstrates GPER1 to interact with aldosterone. The problem remains, through which receptor aside from MR might aldosterone stimulate signaling, is this a GPCR and how does this modulate fibrosis?

Here, we develop a model of steroid-induced fibrosis based on *Drosophila melanogaster*. Genetic data reveal that the *Drosophila* GPRC Dopamine-Ecdysone receptor (DopEcR) (reviewed in Petruccelli et al., 2020) is expressed in cardiomyocytes, and is necessary for exogenous aldosterone and insect ecdysone to induce excess ECM in heart-associated nephrocytes, and to disrupt fly renal function. We likewise document elevated cardiac-renal fibrosis with age and find that this pathology requires endogenous synthesis of ecdysone and cardiomyocyte DopEcR. Similar requirements are found for *Drosophila* Epidermal growth factor receptor (Egfr) (referred to as dEGFR) in terms of exogenous hormone treatments and endogenous aging. Based on our findings we propose that mammalian homologs of DopEcR may offer a novel entrée to understand fibrotic pathology in humans.

## RESULTS

### Steroid hormones induce renal dysfunction at nephrocytes

**Fig. 1.**
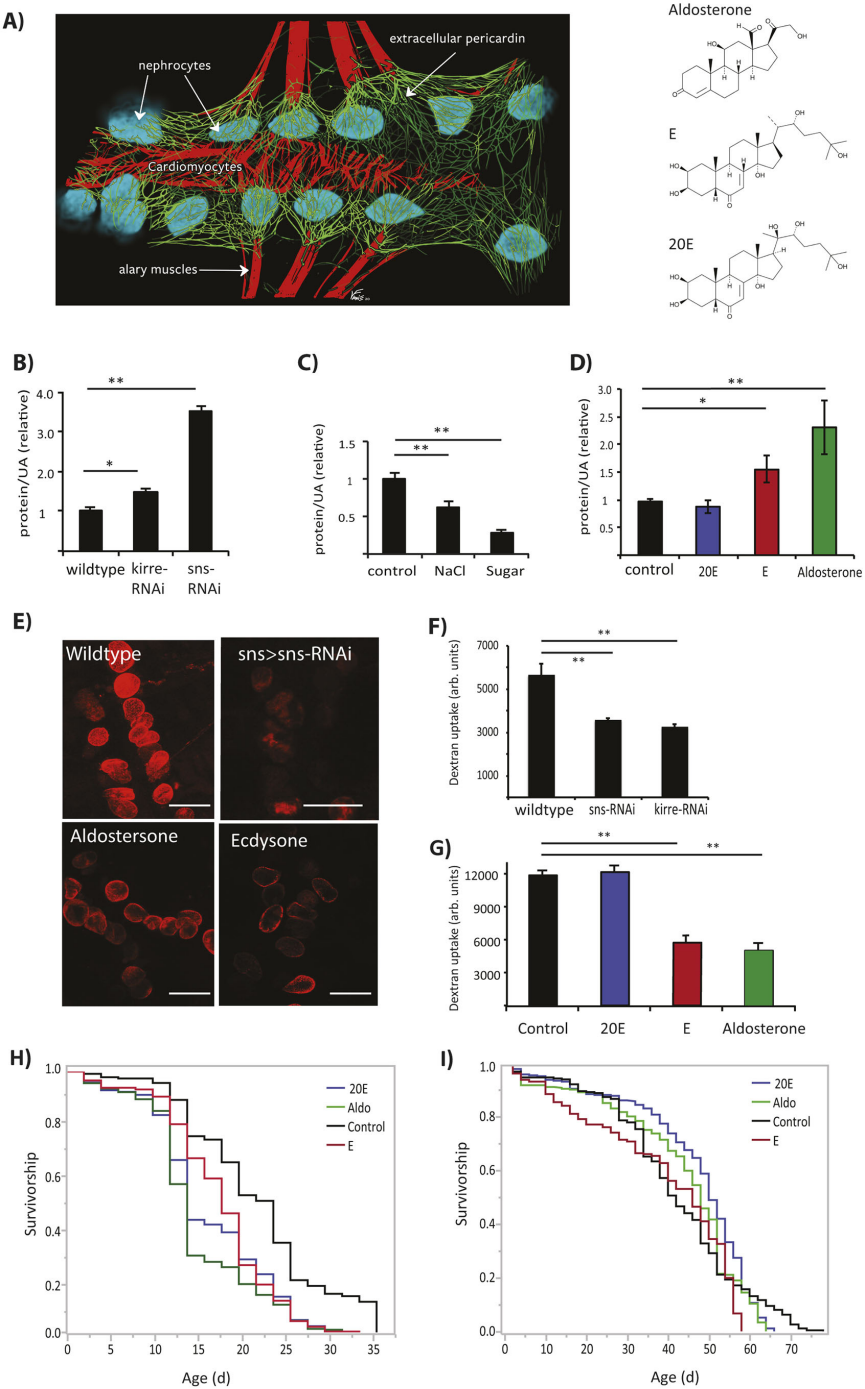
**Aldosterone and ecdysone induce renal dysfunction in adult *Drosophila*.** (A) Heart-renal structure original illustration by Vinald Francis, Brown University, modeled from an image in Rotstein and Paululat (2016) (left): cardiomyocytes within the tubular heart and connective alary muscles, red; surrounding pericardial nephrocytes, blue; cardiac extracellular matrix (ECM) comprised of collagen Pericardin, green. Structures of steroid hormones (right): human aldosterone, and insect ecdysone (E) and 20-hydroxyecdysone (20E). (B) Proteinuria measured as excreted protein/uric acid (UA) in 3-week-old males expressing RNAi in nephrocytes to deplete slit diaphragm proteins encoded by *kirre* or *sns* (each genotype, *n*=6 biological replicates with 15 males each). (C) Proteinuria in 3-week-old old male adults fed a high-salt diet and high-sugar diet; combined data from four independent wild-type backgrounds, each with four biological replicates of *n*=20. Values normalized to control treatment within each background. (D) Proteinuria in 3-week-old males fed 20E, E or aldosterone for 2 weeks; combined data across three wild-type backgrounds, each with four biological replicates of *n*=20. (E) Dextran-bead filtration assay for nephrocyte function; confocal images (representative *z*-stack) of nephrocytes of 3-week-old females. Efficient filtration was seen in wild-type; impaired filtration occurs with depletion of slit diaphragm (*sns*-RNAi) and by treatment of wild-type with aldosterone or E. (F,G) Fluorescence intensity (arb. units, arbitrary units) quantified from biological replicates of nephrocytes from dextran-bead filtration assay when slit diaphragm is depleted by RNAi, and for wild-type adults treated with 20E, E or aldosterone (each genotype, *n*=5). Statistics in B-D, F and G were performed with one-way ANOVA with Dunnett's post hoc comparison to control, *P<0.05, **P<0.01; mean±s.d. (H) Survival upon high-salt diet (1.5% NaCl) for cohorts (each, *n*=230-330) continuously treated with 20E, E or aldosterone relative to control. Survival was significantly reduced by each treatment, pairwise contrasts to control, log-rank test, *P*<0.001. (I) Survival upon normal diet for adults (each cohort, *n*=216-280) continuously treated with 20E, E or aldosterone. Relative to control (median life span=42 days), survival was increased by 20E (median life span=50 days; log-rank test, *P*=0.051), but not significantly affected by aldosterone (median lifespan=48 days, log-rank test, *P*=0.742) or E (median lifespan=46 days, log-rank test, *P*=0.185). Scale bar: 100 μm.

The tubular heart of adult *Drosophila* is lined by pericardial cells, podocyte-like nephrocytes that conduct size-selective filtration of hemolymph (Beyenbach et al., 2010; Weavers et al., 2009) (Fig. 1A). The heart tube and associated nephrocytes are enmeshed in an ECM composed of collagen-like proteins including Pericardin (collagen IV) (Chartier et al., 2002; Hollfelder et al., 2014). In a first step to develop a model of *Drosophila* renal fibrosis, we measured protein in adult excreta (frass) as an analog to proteinuria seen in humans with glomerular dysfunction (Ziyadeh and Wolf, 2008). Frass is a by-product of both digestion and discharge from renal Malpighian tubules, gut-associated structures that maintain ionic and water balance (Beyenbach et al., 2010; Weavers et al., 2009; Zhuang et al., 2009; Na et al., 2015). Previous work shows that the appearance of frass can be modulated by diet, mating and internal metabolic state (Cognigni et al., 2011), and by the activity of heart-associated nephrocytes (Zhang et al., 2013a; Helmstädter and Simons, 2017). We asked whether frass protein content could be affected by nephrocyte function. We collected frass from adult males (to exclude eggs) in microcentrifuge tubes and measured total protein content, normalized to uric acid as a way to account for excretion volume. To manipulate nephrocyte function, we depleted nephrocyte slit diaphragm genes *kirre* and *sticks-n-stones* (*sns*), which encode homologs of mammalian nefrin. Previous reports show that reduced *kirre* and *sns* impairs nephrocyte filtration measured by uptake of fluoro-dextran beads (Weavers et al., 2009; Na et al., 2015). We replicated this result (Fig. 1E,F) and observed that reduced *kirre* and *sns* also elevated protein excretion (Fig. 1B). Thus, defects in nephrocyte function can induce proteinuria in *Drosophila*.

We next assessed how frass protein content was affected by nutrient and physiological conditions as occurs with human chronic kidney disease. Diets of high sugar or salt decreased protein excretion compared to normal diet (Fig. 1C), perhaps by altering adult metabolic state. To find a treatment that might increase proteinuria, we fed aldosterone to adult *Drosophila*. Protein in frass was elevated in adults fed aldosterone for 2 weeks (Fig. 1D) but not in those fed aldosterone for only 24 h (Fig. S1). *Drosophila* do not synthesize aldosterone, a mammalian steroid hormone (Fig. 1A) produced in the renal cortex. Rather, aldosterone likely acts in *Drosophila* as a mimic of insect steroids (Fig. 1A), or by providing a precursor for the synthesis of insect steroids. The primary active steroid in *Drosophila* is 20-hydroxyecdyone (20E). 20E is oxidized from the prohormone ecdysone by 20-hydroxylase (encoded by *shade*) at target cells. 20E activates the nuclear hormone Ecdysone receptor (EcR) to modulate transcription. Interestingly, feeding adults 20E for 2 weeks did not stimulate proteinuria, but proteinuria was elevated in adults chronically fed ecdysone (Fig. 1D). Likewise, chronic aldosterone and ecdysone, but not 20E, suppressed dextran filtration by nephrocytes (Fig. 1G). Although only aldosterone and ecdysone affected nephrocyte function and associated proteinuria, all tested steroids (aldosterone, ecdysone and 20E) reduced survival of adults on a high-salt diet (Fig. 1H), indicating that each exogenous hormone has some capacity to impart biological activity. We found no consistent association between exogenous steroids and adult survival on a normal diet (Fig. 1I).

### Elevated ECM drives renal dysfunction

**Fig. 2.**
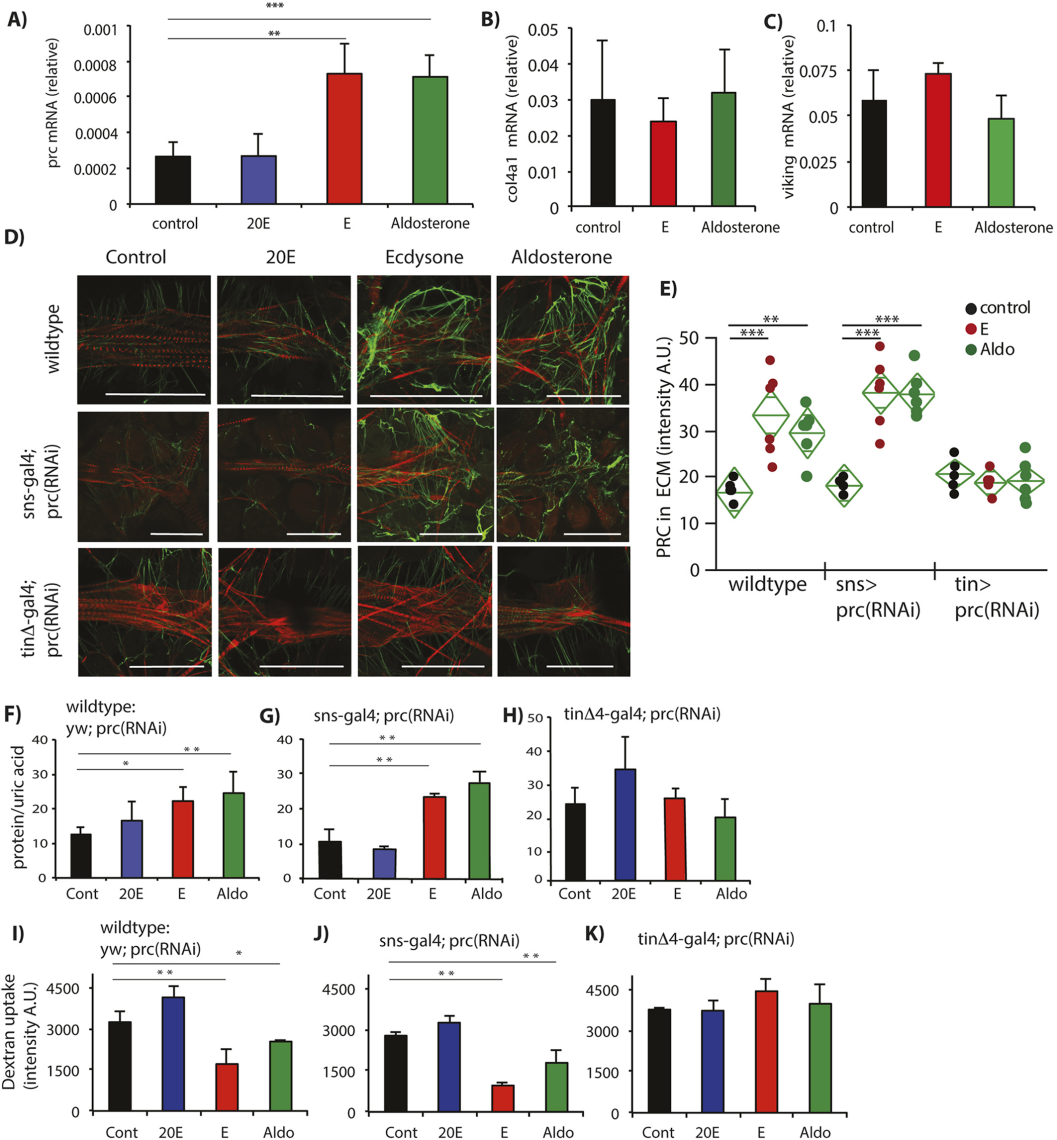
**Pericardin from cardiomyocytes induced by steroids produces renal dysfunction.** (A) *pericardin* (*prc*) mRNA in heart-nephrocyte tissue induced in females fed ecdysone (E) and aldosterone, but not 20-hydroxyecdysone (20E), expressed relative to ribosomal protein L32 (*Rp49*) mRNA from the same sample (each genotype, *n*=5 biological replicates of ten pooled tissues). (B,C) *Collagen-4a1* (*Col4a1*) and *viking* mRNA, expressed relative to *Rp49* mRNA from the same sample, in heart-nephrocyte tissue are not induced by steroid hormones (each genotype, *n*=5 biological replicates of ten pooled tissues). (D) Confocal images (representative *z*-stacks) of heart-nephrocyte tissue of 3-week-old females after a 2-week treatment with 20E, E or aldosterone; wild-type and knockdown genotypes to deplete *prc* mRNA in nephrocytes [*sns*-gal4>UAS-*prc*(RNAi)] and cardiomyocyte [*tin*Δ4-gal4>UAS-*prc*(RNAi)]. Phalloidin (red) stains cardiomyocyte actin; secondary antibody marks PRC protein (green) in ECM around nephrocytes and the heart. (E) Quantification of staining intensity (A.U., arbitrary units) for PRC protein in ECM (each genotype, each treatment, *n*=6). (F-H) Proteinuria in 3-week-old males fed 20E, E or aldosterone for 2 weeks, assessed in wild-type background [yw/UAS-*prc*(RNAi)] (F), and in genotypes that reduce *prc* [UAS-*prc*(RNAi)] in nephrocytes (*sns*-gal4) (G) or cardiomyocytes (*tin*Δ4-gal4) (H); (each genotype, each treatment, *n*=5 biological replicates with 15 males each). (I-K) Quantification of fluorescence intensity from biological replicates of nephrocytes in *ex vivo* dextran-bead filtration assay in 3-week-old males fed 20E, E or aldosterone for 2 weeks, assessed in wild-type [yw/UAS-*prc*(RNAi)] (I), and in genotypes that reduce *prc* [UAS-*prc*(RNAi)] in nephrocytes (*sns*-gal4) (J) or cardiomyocytes (*tin*Δ4-gal4) (K) (each genotype, each treatment, *n*=3). Statistics in A-C and E-K were performed with one-way ANOVA with Dunnett's comparison relative to control, *P<0.05, **P<0.01, ***P<0.001; mean±s.d. Scale bar: 100 μm.

Pericardial nephrocytes and the heart tube are surrounded by ECM made of collagen-like proteins including Pericardin (Fig. 1A), Col4a1 and Viking (Chartier et al., 2002; Hollfelder et al., 2014; Zhang et al., 2013b). Adults fed aldosterone and ecdysone for 24 h induced *pericardin* (*prc*) mRNA in their cardiac-nephrocyte tissue, but not when fed 20E (Fig. 2A). Collagen encoding-transcripts *Col4a1* and *viking* mRNA were not induced by any of these steroids (Fig. 2B,C). Despite induction of *prc* mRNA, overnight steroid feeding itself did not elevate proteinuria (Fig. S1). In contrast, wild-type adults fed aldosterone and ecdysone for 2 weeks had elevated ECM PRC protein around the cardiac-nephrocyte complex (Fig. 2D,E). Depletion of *prc* mRNA from cardiomyocytes [*tin*Δ4-gal4>*prc*(RNAi)] (efficiency in Fig. S2), but not from nephrocytes [*sns*-gal4>*prc*(RNAi)], blocked the ability of aldosterone and ecdysone to induce excess PRC deposition (Fig. 2D,E). We also determined that *prc* expression in cardiomyocytes was necessary for aldosterone and ecdysone to induce proteinuria and to repress nephrocyte filtration: depletion of *prc* mRNA from cardiomyocytes blocked the ability of aldosterone and ecdysone to induce pathology, whereas depletion of *prc* mRNA in nephrocytes did not (Fig. 2F-K). In contrast, exogenous 20E continued to produce no effects on fibrosis or nephrocyte function, independent of *prc* knockdown (Fig. 2D,F-K). Thus, cardiomyocytes appear to be the source of PRC protein that accumulates in response to chronic exposure to aldosterone and ecdysone, and impairs nephrocyte function.

### The GPCR DopEcR is required for steroids to drive fibrosis

**Fig. 3.**
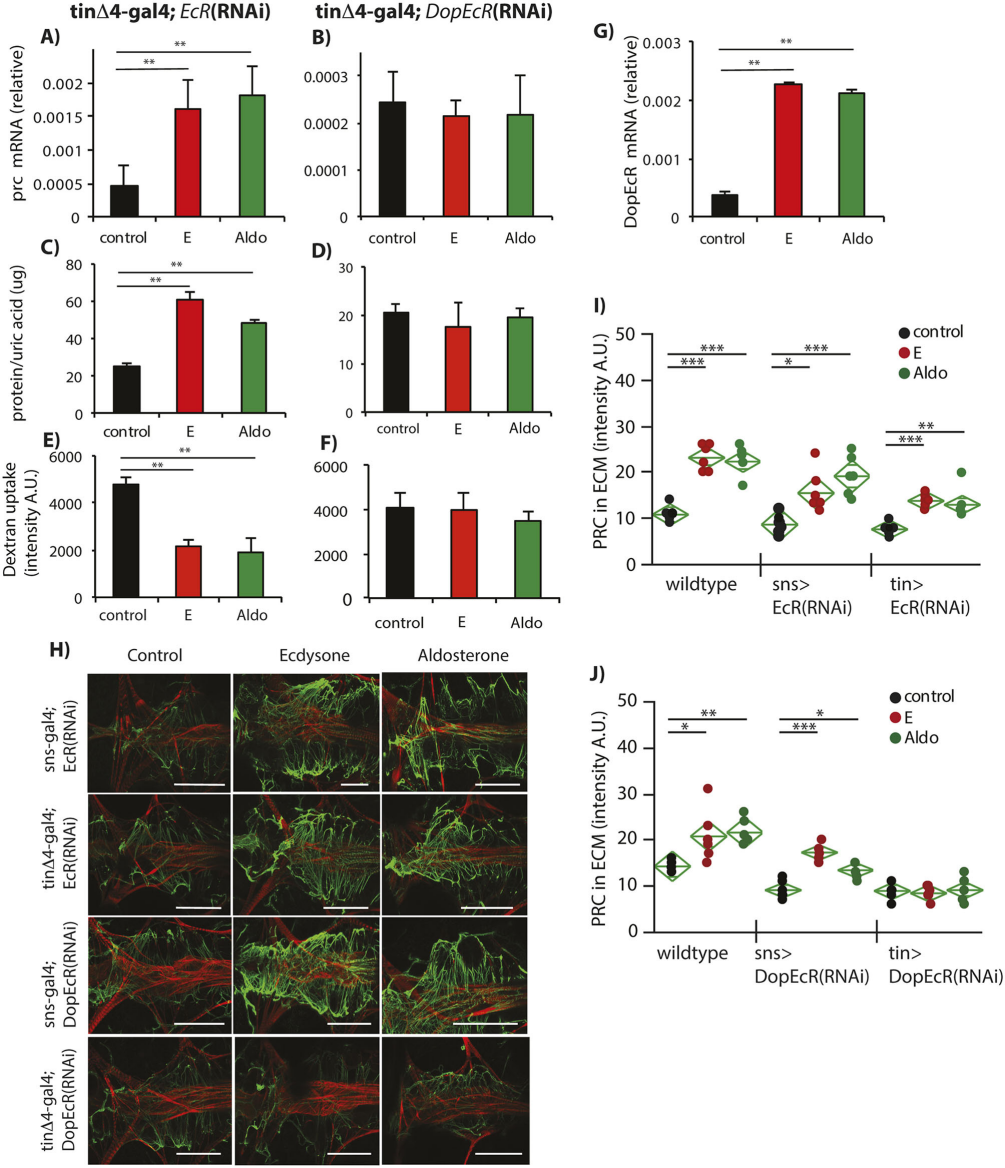
**Cardiomyocyte *DopEcR* is required for steroid induction of fibrosis and renal pathology.** Depletion of nuclear hormone receptor EcR by RNAi did not block ability of ecdysone and aldosterone to induce (A) increases in heart-nephrocyte *prc* mRNA, relative to *Rp49* (each genotype, *n*=5 biological replicates of ten pooled tissues); (C) increases in proteinuria (each treatment, *n*=3 biological replicates with 15 males each); (E) and reduced nephrocyte filtration (each treatment: *n*=3). Depletion of GPCR DopEcR by RNAi blocked the ability of ecdysone and aldosterone to induce (B) increased heart-nephrocyte *prc* mRNA, relative to *Rp49* (each genotype, *n*=3 biological replicates of ten pooled tissues); (D) increased proteinuria (each treatment, *n*=4 biological replicates with 15 males each); and (F) reduced nephrocyte filtration (each treatment: *n*=3). (G) *DopEcR* mRNA, relative to *Rp49,* is elevated in heart-nephrocyte tissue of 3-week-old adults treated overnight with ecdysone (E) or aldosterone (each treatment, *n*=3 biological replicates of ten pooled tissues). (H) Confocal images (representative *z*-stacks) of heart-nephrocyte tissue from 3-week-old females after a 2-week treatment with E or aldosterone, with genotypes to deplete *EcR* or *DopEcR* mRNA in nephrocytes (*sns*-gal4) or cardiomyocytes (*tin*Δ4-gal4). Cardiomyocyte actin stained by phalloidin, red; PRC protein of ECM, green. (I,J) Quantification of PRC staining intensity (A.U., arbitrary units) (each genotype, each treatment, *n*=6), with genotypes to deplete *EcR* mRNA (I) or *DopEcR* mRNA (J) in nephrocytes (*sns*-gal4) or cardiomyocytes (*tin*Δ4-gal4). Statistics in A-G, I and J were performed with one-way ANOVA with Dunnett's comparison relative to control, *P<0.05, **P<0.01, ***P<0.001; mean±s.d. Scale bar: 100 μm.

It is striking that ecdysone but not 20E induces PRC expression and associated renal pathology in *Drosophila*. This suggests that PRC protein in the ECM can be regulated independently of EcR, the canonical nuclear hormone ecdysone receptor of 20E. Indeed, depletion of *EcR* by RNA interference (RNAi) in cardiomyocytes did not prevent the steroid-dependent induction of *prc* mRNA (Fig. 3A), or associated ECM accumulation (Fig. 3H,I) and renal pathology (Fig. 3C,E).

An alternative avenue for action involves DopEcR (CG18314), a membrane GPCR of ecdysone that has been described in the fly brain (Petruccelli et al., 2016, 2020; Ishimoto et al., 2013; Lark et al., 2017). We detected *DopEcR* mRNA in adult cardiac-nephrocyte tissue, and more so in adults fed aldosterone and ecdysone (Fig. 3G). Consistent with a model in which DopEcR is required for aldosterone and ecdysone to stimulate renal pathology, cardiomyocyte-specific knockdown of *DopEcR* [via *tin*Δ4-gal4>*DopEcR*(RNAi)] blocked the ability of aldosterone and ecdysone to induce *prc* mRNA expression (Fig. 3B), elevate proteinuria and inhibit nephrocyte filtration (Fig. 3D,F). Likewise, *DopEcR* in cardiomyocytes is required for aldosterone and ecdysone to induce excess PRC protein (Fig. 3H,J). In contrast, while elevated deposition of PRC was prevented by cardiac-specific knockdown of DopEcR, PRC was not inhibited in flies with nephrocyte-specific *DopEcR* or *EcR* knockdown (Fig. 3H-J).

**Fig. 4.**
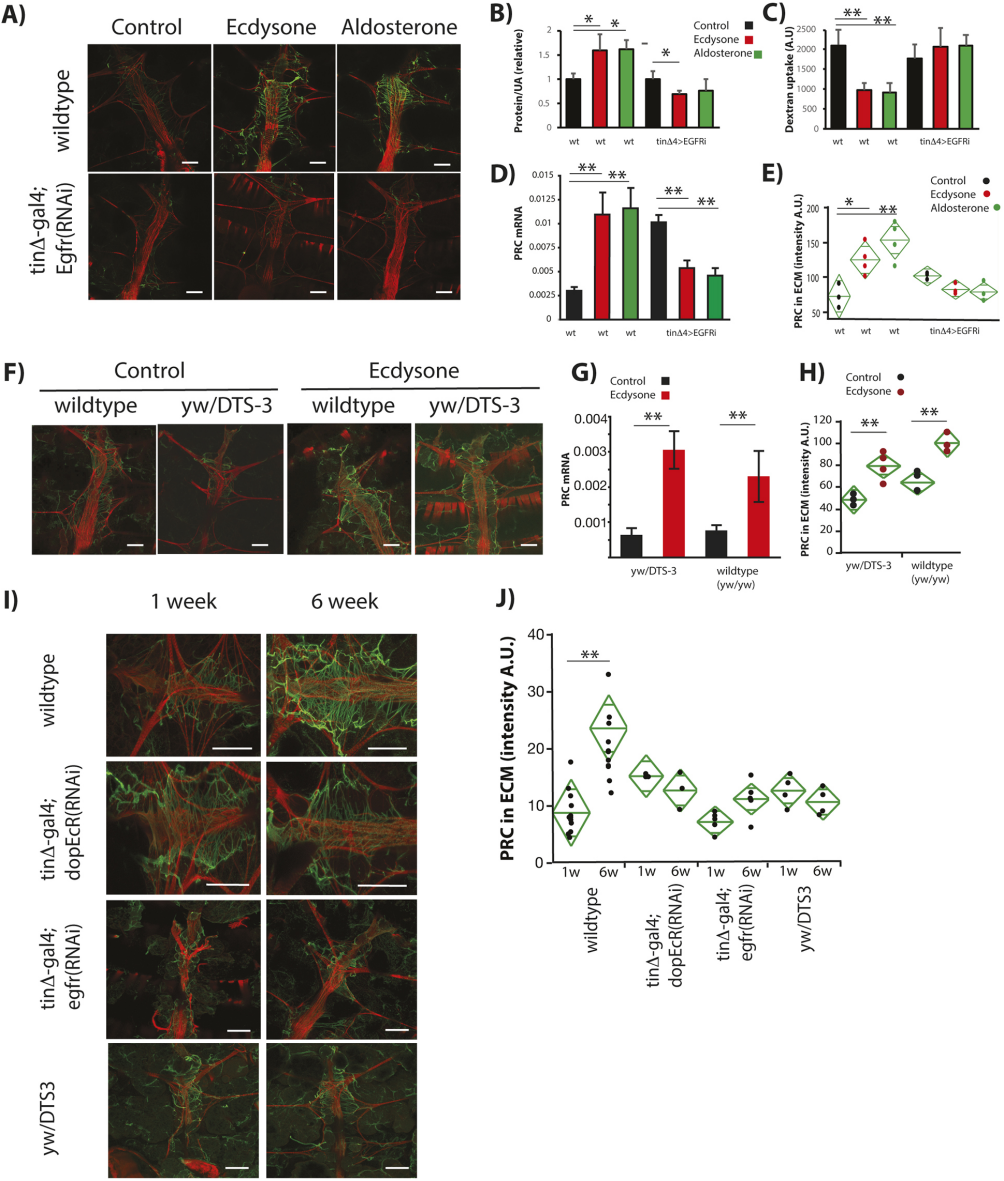
**Ecdysone and aldosterone require dEGFR in cardiomyocytes to induce ECM** **PRC.** (A) Representative *z*-stack confocal images of hearts from wild type (top row) and *DopEcR* knockdown hearts (bottom row). (B) The level of proteinuria in wild-type (wt) flies was increased by ecdysone feeding but showed no increase in flies with RNAi-mediated cardiac knockdown of *dEGFR* (each treatment, *n*=5 biological replicates with 15 males each). UA, uric acid. (C) Dextran filtration as a measure of nephrocyte function was reduced in flies fed ecdysone or aldosterone; this reduction was blocked by cardiac *dEGFR* knockdown. (D) *prc* mRNA, relative to *Rp49*, was induced by ecdysone or aldosterone feeding (each genotype, *n*=3 biological replicates of ten pooled tissues). (E) Quantification of PRC staining intensity, with genotypes to deplete *dEGFR* mRNA from cardiomyocytes (*tin*Δ4-gal4) (each genotype, each treatment, *n*=4). (F-H) Pericardin in ECM: (F) confocal images from control and ecdysone-treated wild-type and *yw*/*DTS-3* females, with (G) *prc* mRNA relative to *Rp49* (each genotype, each treatment, *n*=3 biological replicates of ten pooled tissues), and (H) PRC intensity quantified (A.U., arbitrary units) (each genotype, each treatment, *n*=4). Statistics in B-E, G and H were performed with one-way ANOVA with Dunnett's post hoc comparison relative to genotype control, *P<0.05, **P<0.001; mean±s.d. (I,J) PRC in the ECM increases with age (between 1 and 6 weeks) without exogenous hormone treatments. Age-associated fibrosis is prevented in the *DTS-3* mutant, and when *DopEcR* or *dEGFR* is knocked down in cardiomyocytes (each knockdown genotype, each age, *n*=4; all wild-type controls combined, each age, *n*=10); one-way ANOVA with Dunnett's comparison relative to 1-week-old wild type, **P<0.01; mean±s.d. Scale bars: 100 μm.

Petruccelli et al. (2016) and Ishimoto et al. (2013) demonstrated that DopEcR promoted ethanol sensitivity through suppression of epidermal growth factor receptor/extracellular signal-regulated kinase signaling (EGFR/ERK) in the brain. We asked whether dEGFR was required for ecdysone and aldosterone to induce fly renal fibrosis. Knockdown of *dEGFR* by RNAi in cardiomyocytes prevents the development of renal pathology and fibrosis in adults treated with either hormone (Fig. 4A-E).

Our work suggests that ecdysone acts as an agonist of DopEcR in the heart, where receptor activation modulates organ fibrosis. Nevertheless, it is possible that treatment with exogenous ecdysone antagonizes production of endogenous steroids (ecdysone or 20E), and that this loss promotes fibrosis. If true, knockdown of endogenous ecdysone should itself promote fibrosis, and addition of exogenous ecdysone should not further increase fibrosis. To test this hypothesis, we employed a mutant of the nuclear zinc finger protein encoded by *molting defective* (*DTS-3*; also known as *mld*) (Neubueser et al., 2005), which inhibits the transcription of enzymes required for endogenous ecdysone synthesis. *DTS-3* flies grow and emerge normally at 18°C, and adults switched to 29°C produce little ecdysone. We grew cohorts of *DTS-3* females and control wild-type females following these temperature regimes. As expected, control wild-type females showed little *prc* mRNA and PRC until treated with exogenous ecdysone (Fig. 4F-H). However, contrary to the hypothesis, *DTS-3* females, with anticipated low endogenous levels of ecdysone, also had low levels of *prc* mRNA and PRC until stimulated by exogenous ecdysone (Fig. 4F-H). Exogenous ecdysone appears to positively promote fibrosis rather than act by repressing production of endogenous steroids.

### Renal fibrosis naturally occurs with age and is modulated by ecdysone and DopEcR

To this point we had induced fibrosis by treating flies with external steroids, begging the question, what is the physiological relevance in fibrosis of endogenous ecdysone acting through DopEcR and dEGFR? Endogenous ecdysone is normally elevated in aging *Drosophila*, where whole-animal titers increase several fold between young and aged flies (Zheng et al., 2018). In addition, Vaughan et al. (2018) found the collagen Viking accumulates in *Drosophila* cardiac ECM with age. We therefore measured PRC protein in the heart-nephrocyte ECM of untreated young and aged flies. PRC increased ∼2-fold in 6-week-old flies relative to young adults (Fig. 4I,J). This age-dependent fibrosis was prevented by knockdown of endogenous ecdysone synthesis in adults, using the *DTS-3* system as above (Fig. 4I,J). Likewise, knockdown of both *DopEcR* and *dEGFR* in cardiomyocytes prevented fibrosis in the aged flies (Fig. 4I,J). These results suggest that PRC accumulation in the nephrocyte and cardiac-associated ECM is an intrinsic property of aging flies promoted by endogenous ecdysone acting through cardiac DopEcR and EGFR.

## DISCUSSION

Mammalian aldosterone is synthesized from cholesterol in the adrenal cortex as a 21-carbon, C21-hydroxyl steroid to control plasma Na^+^ and K^+^, water balance and blood pressure. Insect ecdysone is a 27-carbon steroid with hydroxyl groups at C21 and C27 (Fig. 1A). Adult *Drosophila* produce ecdysone in ovaries and several somatic tissues including the Malpighian tubules (Zheng et al., 2018; Belles and Piulachs, 2015). Circulating ecdysone is converted at target cells to 20E, which induces transcriptional programs by activating the nuclear hormone EcR. Our data show that exogenous aldosterone and ecdysone, but not 20E, stimulate deposition of PRC in adult heart-nephrocyte ECM acting through the GPCR DopEcR and not the canonical nuclear hormone receptor EcR. How aldosterone mimics ecdysone in this context remains unknown. Work is needed to determine if aldosterone has affinity to DopEcR, or if aldosterone acts as a precursor molecule that can be converted to ecdysone within *Drosophila*. We likewise do not understand why exogenous 20E does not stimulate fibrosis whereas ecdysone produces a strong response. Previous work found that 20E and ecdysone have affinity for DopEcR in isolated Sf9 cell membranes (Srivastava et al., 2005), while exogenous 20E modulates DopEcR activity measured from fly brain cAMP levels, by brain nicotine-induced Ca^2+^ responses and by adult behavior (Petruccelli et al., 2016; Ishimoto et al., 2013; Lark et al., 2017). It also remains to determine what roles ecdysone plays in the regulation of PRC in the heart during normal development; perhaps, we suggest, it facilitates cardiac remodeling during molt and pupation (Wilmes et al., 2018).

Ecdysone circulating in adult hemolymph may act at many sites aside from EcR in fat body and ovary (Schwedes et al., 2011), or from DopEcR in the fly brain (Petruccelli et al., 2016; Ishimoto et al., 2013). Our genetic results indicate that *DopEcR* message is required specifically in cardiomyocytes to modulate steroid-induced fibrosis. Using newly emerging tools well suited to study GPCR in *Drosophila*, we anticipate that future work can directly identify which cells in the fly heart produce functional DopEcR proteins (Kondo et al., 2020; Koles et al., 2015). Fibrosis in human hearts arises from myofibroblasts that secrete ECM proteins including fibronectins, elastins and collagens (Tuttle et al., 2014; Meran and Steadman, 2011; Mack and Yanagita, 2015). Based on these parallels, we propose that *Drosophila* cardiomyocytes and mammalian myofibroblasts have analogous functions to produce ECM.

We find that chronic induction of *prc* by steroid hormones stimulates excess PRC protein in the ECM surrounding the myocardial-nephrocyte cells, induces proteinuria and inhibits nephrocyte filtration. Excess heart-associated ECM was previously reported in aged *Drosophila*, measured by accumulation of PRC and the collagen subunit Viking (Vaughan et al., 2018). Here, we also find that PRC increases in cardiac-nephrocyte ECM of old females. Remarkably, systemic knockdown of adult ecdysone synthesis, which otherwise increases with age (Zheng et al., 2018), prevents elevated PRC in aged females, as does cardiomyocyte knockdown of DopEcR (and EGFR). From our observation that steroids elevate *prc* mRNA, we propose that DopEcR promotes fibrosis during aging by inducing *prc* mRNA, and subsequent translation and secretion of PRC, rather than by modulating ECM breakdown.

DopEcR is a dual agonist receptor (Evans et al., 2014). In neurons, DopEcR transduces signals from both dopamine and ecdysone to regulate mating behavior and ethanol sensitivity (Petruccelli et al., 2016; Ishimoto et al., 2013). Activation by dopamine induces cAMP-mediated signal transduction. Ecdysone has greater affinity to DopEcR than does dopamine, and through unknown mechanisms will displace dopamine and induce alternative signal transduction mediated by MAP kinases (Srivastava et al., 2005; Lark et al., 2017). Reports are mixed on whether ecdysone also affects cAMP via DopEcR because dopamine alone can increase cAMP in Sf9 cells expressing DopEcR (Ishimoto et al., 2013; Srivastava et al., 2005). In mammalian cells, cAMP can induce PKA (also known as PRKACA) to phosphorylate CREB (also known as CREB1), which then localizes to promoters. Human CREB targets include several collagen genes, and cAMP stimulation suppresses collagen-I expression in a CREB-dependent manner (Zhang, 2005, 2005; Impey et al., 2004). Accordingly, we hypothesize that dopamine-cAMP-associated transduction initiated from DopEcR may negatively regulate *prc*.

In contrast to the potential action of dopamine, DopEcR stimulated by ecdysone can signal through dEGFR to ERK1/2 as seen in transfected Sf9 cells and in a neuronal analysis of ethanol-induced sedation (Petruccelli et al., 2016; Srivastava et al., 2005). We now find that myocardial *dEGFR* is also required for steroids to induce PRC and nephrocyte dysfunction, and for PRC to accumulate with age. In humans, EGFR signaling is a crucial regulator of fibrosis (Zhuang and Liu, 2014; Rayego-Mateos et al., 2018). The EGFR ligands TGF-α and epidermal growth factor are expressed in kidney cells in which activated EGFR stimulates ERK1/2, Janus kinase/signal transducers and activators of transcription, and PI3 kinase/AKT. In renal interstitial fibrosis, EGFR regulates TGF-β1 via ERK1/2 to activate myofibroblasts and promote expression of ECM collagens (Liu and Zhuang, 2016). Notably, EGFR can be transactivated independent of its extracellular ligands, including by the activity of GPCRs such as the angiotensin II receptor, and this action is mediated intracellularly by the sarcoma kinase Src. Furthermore, Src-mediated transactivation has been shown to accentuate renal fibrosis in mammals (Yan et al., 2016; Wang, 2016; Qian et al., 2016). Based on our current observations, we hypothesize that ecdysone-stimulated DopEcR might stimulate Src to facilitate ligand activation of dEGFR [*Src42A* (Cordero et al., 2014)].

Studies in mammals suggest that aldosterone could also signal via a membrane-associated GPCR. GPER1 has been proposed to function as a non-genomic aldosterone receptor and as a potential homolog of DopEcR (Evans et al., 2016; Barton and Meyer, 2015; Feldman and Limbird, 2015). GPER1-dependent induction by aldosterone is reported in renal cortical adenocarcinoma cells (Feldman et al., 2016), and in mouse models with tissue-specific mineralocorticoid receptor gene deletion (Lother et al., 2015). However, no data establish a mechanism of non-genomic action for aldosterone through GPER1 (Cheng et al., 2014; Rigiracciolo et al., 2016), and the current steroid candidate for GPER1 is 17β-estradiol (Revankar et al., 2005). Using the DIOPT Ortholog Prediction Tool, we identified several potential alternatives for the DopEcR homolog in the human genome including GPR52 (sequence similarity 46%) and UTS2R (sequence similarity 44%). GPR52 is an orphan GPCR described to modulate huntingtin protein (HTT) through cAMP-dependent mechanisms (Yao et al., 2015). Knockdown of *Gpr52* reduces HTT levels in a human tissue model, whereas neurodegeneration is suppressed by knockdown of *DopEcR* in *Drosophila* that express human *HTT*. The urotensin II receptor (UTS2R) is a conserved GPCR implicated in renal fibrosis by trans-modulating EGFR and activating MAPK protein (Tian et al., 2008; Vaudry et al., 2015). The kidneys of diabetic rats express elevated urotensin II, and UTS2R is required for exogenous urotensin to induce TGF-β1 and collagen in the renal ECM. If functional homology can be established between DopEcR and these mammalian candidates, *Drosophila* will provide a new model system to uncover mechanisms of fibrosis in humans.

## MATERIALS AND METHODS

### Fly stocks

Unless noted, wild-type flies were *yw* (ywR). *tin*Δ4-gal4 was a gift from the Manfred Frasch laboratory (Lo and Frasch, 2001). *sns*-gal4 was obtained from the Bloomington *Drosophila* Stock Center (#76160) and UAS-*prc*(RNAi) was obtained from the Vienna *Drosophila* Research Center (VDRC) (#GD41321). UAS-*EcR*(RNAi) was from the laboratory of Neal Silverman (University of Massachusetts Medical School, Worcester, MA, USA). *DopEcR*(RNAi) (kk103494) and *dEGFR*(RNAi) (kk100051) were also obtained from VDRC. Except to measure proteinuria, all assays were conducted with females because their larger size facilitates dissection.

### Steroid and diet treatment

Ecdysone (Sigma-Aldrich, #E9004), 20E (Sigma-Aldrich, #H5142) and aldosterone (Sigma-Aldrich, #A9477) were dissolved in ethanol at 5 mg/ml. Flies were reared in bottles with emerging adults permitted to mate for 2-3 days. Adult were then separated by sex into 1-l demography cages with ∼120 adults per cage. Adults were fed a standard laboratory cornmeal-yeast-sugar diet until 7-10 days of age, at which time the food medium was switched to 0.5 g Genesee Scientific instant fly medium (Genesee Scientific, #66-117) hydrated with 2 ml water containing vehicle control (150 μl ethanol) or vehicle with 150 µl hormone solution. For chronic exposure to steroids, flies were treated for the next 14 days at 25°C fly with media vials changed every 3 days. For overnight exposure to steroids, flies were maintained in demography cages with untreated instant fly medium until 20 days of age, then exposed to diets with appropriate hormone conditions for 24 h. In all trails, renal traits and *prc* mRNA were assessed in adults at 3 weeks of age. The same protocols were used to expose adults to high salt or high sugar, and instant medium was moistened with water containing 1.5% NaCl. To vary dietary glucose, adults were aged to 3 weeks on an otherwise standard laboratory diet with glucose was set at 5% (control, normal) or at 34% (high-sugar diet).

### Proteinuria

For each biological replicate, frass of 15 males was collected for 2.5 h in a 1.5-ml centrifuge tube covered with a breathable foam plug, at 25°C. Males were used in this assay to avoid complications of eggs also being laid in the tubes by females. Deposited frass was fully dissolved with 20 µl 1×PBS, providing 10 µl to assess total protein and 10 µl to measure uric acid, which serves as a proxy for the quantity of deposited frass. Total urine protein was determined by Pierce BCA Protein Assay (Thermo Fisher Scientific, #23227). Uric acid was measured by QuantiChrom Uric Acid Assay (Bioassay Systems, DIUA-250).

### Immunohistology

Nephrocyte-heart tissue from 3-week-old females was dissected in PBS, fixed with 4% formaldehyde in PBS for 30 min and washed three times for 10 min with PBTA (1×PBS, 1.5% bovine serum albumin, 0.3% Tween 20) at room temperature. The washed tissue was incubated with 100 µl primary antibody (mouse anti-PRC, 1:100; Developmental Studies Hybridoma Bank) diluted in PBTA overnight at 4C, washed 3×10 min with 1 ml PBTA at room temperature, then incubated in secondary antibody (goat anti-mouse Alexa Fluor 488, 1:200; Alexa Fluor 555-phalloidin, 1:100; Thermo Fisher Scientific) diluted in PBTA overnight, washed 3×10 min with 1 ml PBTA at room temperature and mounted. Confocal images were obtained with a Zeiss 800 and quantified by ImageJ software. The full length of the heart tube, pericardial cells and associated ECM network was imaged from all samples at 488 nm with the same laser intensity setting to produce a *z*-stack comprised of 46 optical slices.

### Nephrocyte filtration

Adult nephrocyte-heart tissue was dissected in artificial *Drosophila* hemolymph (ADH; 108 mM Na^+^, 5 mM K^+^, 2 mM Ca^2+^, 8 mM MgCl_2_, 1 mM NaH_2_PO_4_, 4 mM NaHCO_3_, 10 mM sucrose, 5 mM trehalose, 5 mM Hepes, pH 7.1), incubated at 25°C for 15 min with Alexa Fluor 568-Dextran (10,000 MW, Life Technologies) diluted in ADH at a concentration of 0.33 mg/ml, washed 3×10 min with cold PBS at 4°C, then fixed in 4% formaldehyde for 10 min at room temperature, washed 3×10 min with PBS at room temperature and mounted in PBS. Confocal images were obtained with a Zeiss 800 and quantified by ImageJ software.

### Quantitative RT-PCR

Total RNA was extracted from dissected renal-cardiac tissue in Trizol reagent (Invitrogen, Grand Island, NY, USA) and treated with DNase (Ambion). DNase-treated total RNA was quantified with a NanoDrop ND-1000. cDNA was synthesized using an iScript cDNA synthesis kit (Bio-Rad) and measured on an ABI prism 7300 Sequence Detection System (Applied Biosystems, Carlsbad, CA, USA). Three to five biological replicates were used for each experimental treatment (specifics in figure legends). In all figures, mRNA abundance for each gene is expressed relative to ribosomal protein L32 (*Rp49*; also known as *RpL32*) mRNA from the same sample by the method of comparative CT. Primers for qPCR are provided in Table S1.

## Supplementary Material

Supplementary information
