# Supplementary material for: Extracellular matrix induced by steroids and aging through a G-protein-coupled receptor in a Drosophila model of renal fibrosis
Source: Dis Model Mech. 2020 Jun 24;13(6):dmm041301. doi: 10.1242/dmm.041301 (PMC7328168; doi:10.1242/dmm.041301)
Supplement: Supplementary information [file dmm-13-041301-s1.pdf]

Fig. S1. Overnight feeding of aldosterone to 3 week-old males did not increase proteinuria.

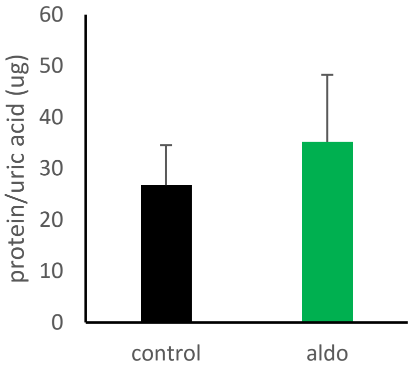

Fig S2. Validation of RNAi efficiency in cardiomyocytes, driven by tinΔ4-gal4. Mean expression levels were determined by qPCR (+/- SD, normalized to *rp49*) from heart-nephrocyte tissue; dissected from 20 day old females; three replicates per genotype. All differences significant at $p < 0.02$.

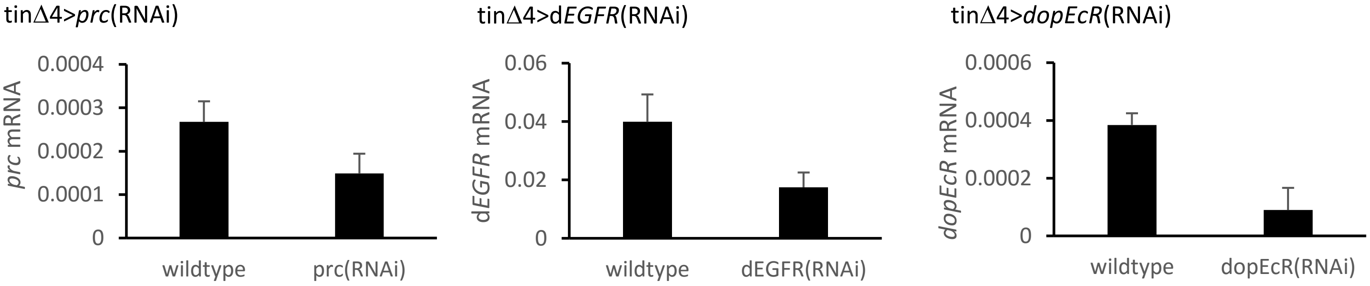

Table S1. Primers for qPCR

	Forward primer	Reverse primer
**Rp49**	5’-GCA CTC TCT GTT GTC GAT ACC CTT G-3’	5’-AGC GCA CCA AGC ACT TCA TC-3’
**Pericardin**	5’-CGG AGG ACA GGC TAC AAT AAG-3’	5’-TTC CAG GCT GAG TTT CGT ATC-3’
**Col4a1**	5’-GCT CTG TGC GAT TTG AGT TTG-3’	5’-CTT CTG CTC CCT TGA ATC CTT-3’
**Viking**	5’-GAT CTA CGA CAA CAC TGG TGA G-3’	5’-TTC GCC ACG AAG TCC AAT AG-3’
**EcR**	5'-TGA AGA CTC CTA TGC TGC-3'	5'-CGA CGT TGT GCT TCG TAA-3'
**dopEcR**	5’-CTT AGG TCC CAG CCT CAT TTC-3’	5’-AGC CAG AGC AGT TGC ATA TT-3’

Disease Models & Mechanisms • Supplementary information
